# Navigating Adversity: Non-Completion and Evacuation of Trail Runners at the 2022 Magoebaskloof Forest Marathon

**DOI:** 10.17159/2078-516X/2026/v38i1a23943

**Published:** 2026-04-15

**Authors:** TE Maleka, A Jansen Van Rensburg, C Viljoen, G Ramkilawon, DA Ramagole, A Nair, DC Janse van Rensburg

**Affiliations:** 1 Section Sports Medicine and SEMLI, Faculty of Health Sciences, University of Pretoria, Pretoria, South Africa; 2 Department of Physiotherapy and SEMLI, Faculty of Health Sciences, University of Pretoria, Pretoria, South Africa; 3 Department of Statistics, Faculty of Natural and Agricultural Sciences, University of Pretoria, South Africa; 4 Medical Advisory Panel, World Netball, Manchester, UK

**Keywords:** did not finish (DNF), trail marathon, medical evacuation

## Abstract

**Background:**

Trail running occurs on natural, uneven terrain. The literature is limited in exploring factors that contribute to trail runners not completing a race.

**Objectives:**

This study firstly investigated self-reported reasons for runners who did not finish (DNF) the 2022 Magoebaskloof Forest Marathon across 10km, 20km, 34km, and 50km distances, and secondly assessed runners’ evacuation methods.

**Methods:**

A descriptive cross-sectional study of consenting trail runners (n=429) who completed the post-race DNF survey (10km, 20km, 34km and 50km distances). Within two weeks post-race, runners reported whether they finished, reasons for discontinuing and evacuation methods. We report numbers (n) and point prevalence (%).

**Results:**

Of the 228 runners who completed the post-race survey, 16 (7%) DNF. DNF runners entered the 34 km (19%) and 50 km distance (81%) and were aged 31–50 years (94%). No DNFs occurred in the 10km and 20km races. Approximately 27% did not meet cut-off times, while 19% got lost despite trail directions. Other contributing factors included pre-existing and new injuries (23%), illnesses (15%), and inadequate training (8%). While 50% of the DNF group had >5 years total running experience, only 19% had >5 years of trail running experience. Runners who finished the trail race had significantly more trail running experience than those who DNF (p=0.0082). The primary evacuation method was hiking back to the nearest aid station (63%), with 19% subsequently transported by shuttle.

**Conclusion:**

Multiple factors may have contributed to the race non-completion. Many DNF runners started with injuries or illnesses, inadequate training, or limited trail-running experience. Event planners should improve measures to prevent runners from getting lost. These findings emphasise the importance of preparation, navigation skills, and race management in ensuring trail running safety and success.

Trail running involves navigating natural trails through forests, mountains, deserts, and other scenic landscapes. Unlike conventional road running on paved surfaces, trail running requires runners to navigate diverse, often challenging terrain, including dirt paths, rocky trails, muddy routes, and steep elevation changes.^[1,2]^ This sport has shown significant growth in recent years, as evidenced by the emergence of several long-distance trail running events.^[3]^ Competitive trail running events, ranging from short distances to ultra-marathons, add a challenging aspect to the sport.

The inaugural Magoebaskloof Forest Marathon in Limpopo Province, South Africa, was held on 20 March 2022 and featured 10, 20, 34, and 50km races.^[4]^ Due to the nature of trail running, not all participants complete the race. In both trail and road running, factors such as inadequate fitness, fatigue, underlying illnesses, and acute or chronic injuries can prevent runners from completing their race.^[5,6,7]^ Participants who do not complete the race are classified as “did not finish (DNF)” and are often omitted from the final results. Injuries and medical illnesses are common reasons participants do not finish a race. In a study on personal best marathon performances during a 24-hour run, 32% of the participants DNF due to medical problems.^[7,8]^

The remoteness and rugged terrain characteristic of trail runs make it difficult to provide the level of logistical and medical support typically available in road-running events. These races take place in mountainous, natural environments with uneven terrain and significant elevation changes, limiting vehicle access and restricting medical teams’ reach to participants. As a result, runners often need to walk out of remote sections of the course before assistance can be provided. This means that medical care and the extraction of acutely ill or injured runners can be challenging, frequently requiring participants to self-evacuate even when experiencing illness or injury.^[9]^

In contrast, road running marathon events have been more extensively investigated. Prior research has identified fatigue, undertraining, injuries, and illness as common reasons for not finishing a race.^[10]^ However, the reasons for DNF outcomes in trail running remain poorly reported. Although several studies have reported on the incidence and types of injuries and illnesses in endurance events^[11,12,13]^, limited evidence exists regarding the specific reasons for non-completion, the distance at which runners withdraw, and the methods of evacuation used to assist them.

Given the unique challenges of trail running, such as difficult terrain, environmental exposure, and restricted vehicle access, understanding these factors is crucial. This knowledge can help athletes prepare more effectively, enable event organizers and medical teams to enhance safety protocols, and improve overall race preparedness.

Therefore, the present study aimed to determine the frequency of runners who started but DNF the Magoebaskloof Forest Marathon race and their self-reported reasons for not finishing. It further examines the factors and challenges faced by the DNF group, analysing the evacuation methods used to help runners exit the forest and return to the base, with the aim of offering recommendations on safety, preparedness, and support infrastructure for future trail running events.

## Methods

### Study design and ethical considerations

A prospective cross-sectional study analysed data collected from post-race trail runners within two weeks of the 2022 Magoebaskloof Forest Marathon event. The Research Ethics Committee of the University of Pretoria granted ethical approval (REC: 427/2022). Participants provided informed consent for the use of their data for research purposes. All collected data were coded, kept anonymised, and stored in a secure data repository.

### Setting

**Fig. 1:**
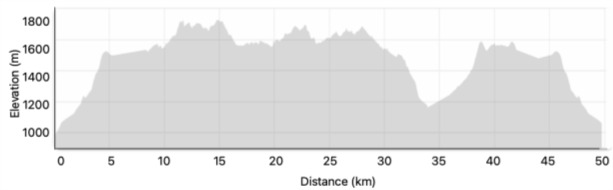
The Magoebaskloof Forest 50km Ultra Marathon track.^[4]^

The forest marathon took place in a subtropical forested and mountainous region of the Limpopo Province, South Africa. Figure 1 below illustrates the course profile of the 50 km race from start to finish. The first 15km are characterised by steep elevation gain, while the following 20km exhibit minimal elevation variability.^[4]^ Notably, a steep descent occurs as participants near the 34km aid station, requiring significant eccentric control and stability.

### Study population

**Fig. 2:**
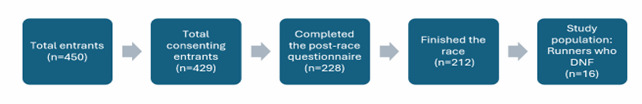
Flow chart illustrating the sampling of the DNF study population for the Magoebaskloof Forest Marathon. DNF, did not finish.

A total of 450 trail runners initially registered and started one of the event’s distance categories (10km, 20km, 34km, or 50km). All participants completed a pre-race questionnaire, and 429 provided consent to take part in the study. Post-race, 228 participants completed the DNF questionnaire. Of these, 212 runners finished the race (206 fully completed the questionnaire, and 6 did not indicate their race outcome but were reasonably assumed to have finished; their responses were considered missing), while 16 runners DNF. Focusing on these participants enabled us to link their injury and illness history to race non-completion ethically. Figure 2 presents a flowchart illustrating the process of sampling the study population.

### Data collection

Runners who started the race completed a pre-race questionnaire, followed by a post-race questionnaire after the event. The focus of this study was to identify and analyse the DNF participants. The pre-race questionnaire enabled the authors to ethically link each runner’s injury and illness history to their race non-completion status, providing a comprehensive understanding of potential contributing factors.

### Online pre-race medical questionnaire

As part of the race entry requirements, all participants (n=450) completed a compulsory online pre-race questionnaire hosted on the Qualtrics^XM^ platform. Designed for clinical use, this self-reported tool serves as a medical screening instrument to identify trail runners at higher risk of developing complications during the race. It has been implemented at multiple trail running events across South Africa, and data collected through its use have been documented in several studies reporting running-related injuries (RRIs) and illnesses.^[13,14]^ During the pre-race screening, data were collected on participants’ demographics (BMI, height, age, weight), total running and trail running experience (in years), clinical details of current injuries (anatomical region, body area, tissue type, and pathology), and illness (affected organ system). Injury occurrence was categorised as acute (occurring within the past two weeks) or chronic (occurring within the past six months) following the 2020 International Olympic Committee (IOC) consensus statement guidelines.^[15]^

### Online post-race medical questionnaire

The DNF trail run questionnaire was developed through a literature review and expert input from sports medicine professionals and trail runners. It was refined for clinical relevance and practicality and implemented digitally. This non-compulsory post-race questionnaire was circulated to all runners to collect DNF information within two weeks after completing the 2022 Magoebaskloof Forest Marathon. Trail runners were required to confirm their registered race distance, indicate whether they started the race, and report their completion status by selecting either “yes” or “no.” Participants reporting a DNF outcome (n=228) were asked to provide the main reasons for discontinuing the race – whether due to illness, injury, or other problems. They were also asked to specify the approximate distance at which they stopped and how they were evacuated back to the finish line.

### Medical aid stations and cut-off times

Medical aid station locations according to race distances:

- 50km: at 9km, 20km, 34km and 41km
- 34km: at 8km and 19km
- 20km: at 9km
- 10km: no aid station

The 50km and 34km races had different starting points, but the first two aid stations were at the same location. The trail race route was a loop, with the start and finish at the same spot. The cut-off times for the various distances were 11 hours (50km), 9 hours (34km), 4 hours (20km) and no cut-off (10km).

### Study outcomes

We report the frequency (n, %) and proportions of demographic variables (age, sex, race distance), running experience (total running experience, trail running experience), and pre-race injury and illness history among all post-race respondents and among male and female runners who DNF the race. We also examine the relationship between male and female race non-completion, considering factors such as the distance at which participants stopped, running duration, incident location, and evacuation mode to the finish line. Additionally, we report the reasons for not finishing the race, including pre-existing conditions, injuries or illnesses sustained during the race, getting lost, or other factors identified by participants.

### Statistical data analysis

The Statistical software R (version 4.2.1; http://www.r-project.org) was used to analyse all data. The data analysis consists of descriptive statistics, such as the mean, standard deviation, frequencies, and proportions, to describe the results. The Chi-squared Goodness-of-Fit Test was used to determine whether our post-race sample was representative of the population of runners who ran the race. Inferential statistics included Fisher’s Exact Test for comparing risk factors between males and females, and Wilcoxon’s Signed Rank Test was for assessing differences in continuous variables between males and females in the DNF group.

## Results

### Participant demographics

A total of 450 trail runners registered for and started the race, of whom 429 (95%) consented to using their pre-race data for research purposes. Of the consenting participants, 228 (53%) runners completed the post-race questionnaire, of whom 16 (9 males, 56%; 7 females, 44%) DNF the race (Table 1). The age and race distances of DNF participants did not differ significantly between males and females (p=0.908 and p=0.932, respectively). The DNF participants only entered the longer races: 34km (32%) and 50km (30%). Most DNF runners entered the 50km race distance (81%) and were between 31–50 years old (94%). The post-race survey participants are representative of all trail run entrants (p >0.05) (Table 1).

### Participants who did not finish

The majority of participants who DNF (n=13; females =5, males =8) entered the 50km race, with most belonging to the 41–50-year age group (n=8), followed by the 31–40-year age group (n=4) (Table 1).

Half (50%) of the DNF trail runners had >5 years of total running experience, which could be defined as the total years a participant has spent regularly participating in running activities, including road and trail running, while only 19% had >5 years of trail running experience Females who DNF had less trail running experience, with 71% having >2 years’ experience, compared to 89% of males (Table 2). A comparison between participants who completed the trail race (n=212) and those who DNF (n=16) showed no significant difference in total running experience (p=0.888). However, a significant difference was observed in total trail running experience, with finishers having greater trail running experience than those who DNF (p=0.008).

### Injury or illness

Among all participants who completed both the pre- & post-race surveys, two runners (0.9%) started the race with a current running injury, while seven runners (3%) started with a current illness. In the six months prior to the race, 28% of all participants reported a RRI.

Within the DNF group, no current RRI were reported. Six participants (38%) reported a previous running-related injury (ankle =3; shoulder =1; lower leg =2). Additionally, one female participant reported a chronic condition (hypercholesterolemia), and one male participant reported a current acute illness (sore throat) (Table 3).

### Distance and time of termination, and method of evacuation

Most DNF athletes (63%) reached approximately 34km into the race, while 38% stopped between 20km and 34km. All participants in the 10km and 20km race categories successfully completed their events. Most DNF participants ran for >9 hours (44%), 7–9 hours (19%) or 5–7 hours (19%). The primary evacuation method varied among participants: 63% ran back to the nearest aid station, 19% used a race shuttle, 6.3% received assistance, and 6% returned independently (Table 4).

### Reasons for not finishing the race

**Fig. 3:**
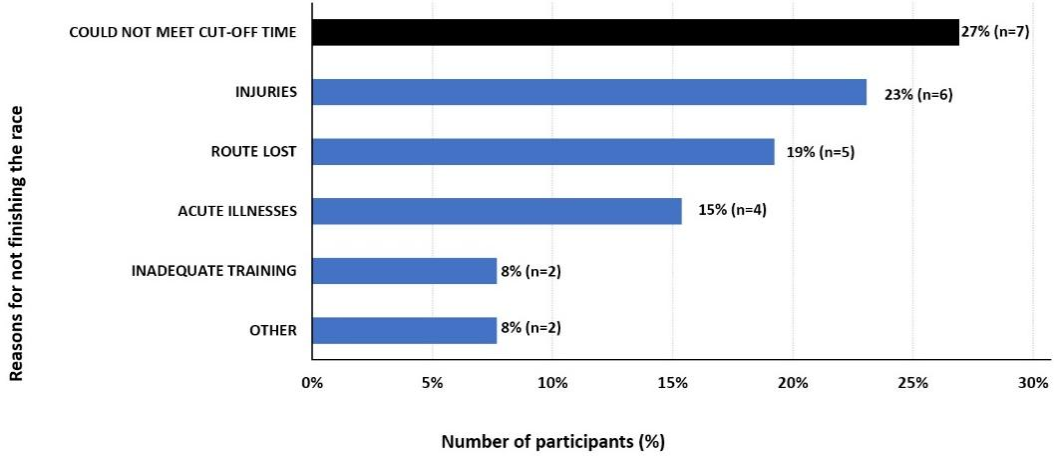
Reasons for not finishing among runners who started the 34 or 50km races. (Participants could give more than one reason).

Participants were allowed to provide multiple reasons for not finishing. Nearly a third (27%) of the participants who DNF the race failed to meet the cut-off times set for the different distances. Six DNF entrants (23%) had pre-existing or acquired injuries during the race, while 19% reported getting lost along the route. Participants also attributed illness (15%) and inadequate training (8%) to their not completing the race (Figure 3).

## Discussion

This study investigated the self-reported reasons of trail runners who DNF a forest marathon and assessed their evacuation methods from the forest. Findings revealed that several factors contributed to non-completion, including a pre-existing injury or illness, inadequate training, and limited trail running experience.

This forest marathon event predominantly attracted middle-aged adults aged 31–50 years, who comprised 70% of all consenting trail runners. This age group also accounted for the majority of DNFs, at 94%. Most DNF participants (n=13; females =5, males =8) entered the 50km race, with the largest proportion in the 41–50-year age group (n=8), followed by the 31–40-year group (n=4). Prior research has suggested that female sex and advanced age may be risk factors for marathon non-completion.^[17,18]^ Our study found that middle-aged athletes were most represented among the DNFs, and there was no statistically significant difference (p=0.908) between the sexes. However, given the small number of participants who DNF, sex-specific risk should be considered exploratory and interpreted with caution. Publications specifically examining female participation in trail running remain limited. Nonetheless, the higher number of female participants in our study underscores the growing trend of increased female participation in endurance sports.^[19,20]^

Among participants who DNF, 50% had >5 years of total running experience; however, only 19% had >5 years of trail running experience. This suggests that general running experience may not fully prepare athletes for the unique challenges of trail races. Interestingly, both male and female DNF participants generally had over two years of total running experience (89% and 86%, respectively). When comparing finishers with DNF participants, there was no significant difference in overall running experience (p=0.888). In contrast, total trail running experience differed significantly, with finishers showing greater trail-specific experience than those who DNF the race (p=0.008). These findings suggest that trail-specific experience, rather than general running experience, may play a more critical role in successful race completion.^[16]^

Most DNFs (63%) were runners with 2–5 years of trail running experience, with a notable majority of females (71%) compared to males (56%). Females (86%) had a comparable total running experience to males (89%), while males had a significantly longer trail running experience of over 2 years (89%). However, despite these results, 50% of all DNFs and 67% of male DNFs had over five years of total running experience. While these patterns align with prior studies suggesting inadequate preparation, poor pacing strategies, and insufficient training for the specific race distance are key contributors to non-completion^[7]^, the limited number of DNFs in our sample prevents firm conclusions, and these trends should be regarded as preliminary.

At study entry, the prevalence of current injuries amongst the total consenting participants was 0.9%, and the prevalence of current illness was 6% in the DNF group. This low figure suggests that most participants likely started the race without acute illness or physical constraints, however the small sample size of DNFs limits confidence in this reasoning.

However, post-race participants’ data showed that 28% of finishers reported a running injury in the past 6 months, compared with 38% in the DNF group. This suggests that pre-existing injuries may contribute to non-completion. Although prior research indicates that injuries and illnesses, whether occurring before or during the race, significantly contribute to not finishing, reinforcing the importance of optimal physical health for race completion,^[21, 22]^ the DNF group displayed a small percentage of current illnesses at study entry (6%).

The primary reasons for not finishing included failure to meet cut-off times (27%), presence of an injury (23%), and getting lost along the route (19%). Emerging evidence suggests that race completion is directly proportional to training volume, underscoring the need for participants to adequately train for race pace and navigation to meet cut-off times.^[7]^ Our findings reinforce the importance of endurance training, proper pacing strategies, and well-planned hydration and nutrition. These knowledge gaps may similarly impact trail runners, affecting their ability to sustain performance over extended periods.

The DNF runners were more prevalent in longer race distances, with 81% of non-finishers entering the 50km race. Dropout rates were higher at key stopping points, notably at 21.7km, 34km, and 42km, suggesting that these segments posed considerable challenges due to sharp descents that demand well-coordinated stability and eccentric muscle function (Figure 1), as well as the steep decline from 35 to 40km.^[23]^ These patterns suggest that steep declines and inclines, together with technical terrain and/or cumulative fatigue, may have contributed to dropouts at this stage of the race.^[24]^ Furthermore, we observed a possible trend of increased withdrawals in the 50km category, particularly when the race duration exceeded six hours, peaking at nine hours. Notably, not finishing occurred at unspecified points in the 34km and 50km distances, indicating potential difficulties in these segments or inadequate preparation. However, given the small number of DNFs, these observations should be viewed as tentative and interpreted with caution.

The remoteness and rugged terrain limited vehicle access, requiring runners to self-evacuate remote sections before assistance could be provided. Among those who reported specific details, withdrawal methods also included shuttle pick-ups and support team assistance. Interestingly, some runners who withdrew managed to walk or run back to the finish line, underscoring their resilience despite the challenging conditions. These findings highlight the importance of race organisers implementing robust support systems, safe evacuation routes, and well-positioned aid stations, particularly in longer, more technical race segments.^[25]^

### Strengths and limitations

The study’s main limitation was the participants’ low response rate (53%) to completion of the post-race survey, which may have led to a biased sample, as the DNF survey was voluntary. Additionally, the reliance on self-reported data introduces the possibility of inaccuracies or subjective interpretation, potentially affecting the reliability of the responses. Recall bias is another concern, as participants may struggle to accurately remember and report the events surrounding their non-completion of the race. Furthermore, the multiple factors contributing to not finishing a race, such as physical, psychological, and environmental influences, may not have been fully captured in a self-reported survey, potentially underestimating the complexity of the reasons behind race abandonment. Although this study offers valuable insights into DNF characteristics in trail running, the findings should be interpreted with caution. The sample consisted of 16 DNF runners from a single event in a subtropical, mountainous region of South Africa, which may not reflect other geographical, environmental, or organisational contexts. Despite these limitations, this study contributes important preliminary data on race non-completion in trail running and highlights the need for multi-event, multi-year research with larger cohorts.

### Recommendations

These findings deepen our understanding of sports participation, especially in trail running, and identify key areas for future research on training, performance, and injury prevention among trail runners. Knowing participants’ motivations and physical abilities is vital, as these factors affect completion rates and can guide improvements in race organisation and support strategies. Future research should gather data from multiple races to enhance representativeness and broader applicability. Collecting information on participants’ motivations, their decision-making processes when withdrawing, and how cut-off times are enforced would provide valuable insights into factors affecting non-completion and race management effectiveness. Furthermore, other factors not yet explored, such as environmental conditions like air temperature, have been shown to significantly influence performance at all levels of running and may also play a role in race non-completion.^[19]^ Including these variables in future studies would help develop a more complete understanding of influencing factors.

## Conclusion

Multiple factors influenced race non-completion. A few of the DNF runners started with injuries or illnesses, inadequate training, and limited trail-running experience. Other key factors contributing to the outcome included age, a running duration of over 9 hours, and a longer race distance, which may have led to higher dropout rates among those who did not finish (81%). Additionally, failure to meet cut-off times and getting lost also contributed to the non-completion of the race among DNF participants. Although runners generally needed to walk out of the more remote areas before assistance could be provided, evacuation was overall managed efficiently, highlighting the event’s preparedness in ensuring runner safety. Despite the low response rate, the application of our study may provide insight and guidance for improving training programs for middle-aged participants, enhancing race-day logistics, and developing support systems to increase completion rates and enhance runner safety among trail runners. It is important to note, however, that the small number of DNFs in this study limits the ability to draw firm conclusions, particularly for subgroup comparisons. Future research with larger samples across multiple events is needed to confirm these preliminary observations.

## Figures and Tables

**Table 1:** Demographic data of all trail run entrants, all post-race study participants and those who DNF

**Characteristics**		**All trail run entrants**	**Participants who completed both pre- and post-race surveys**	**All DNF participants**	**p-value** #

		n=450 (%)	n=228 (%)	n=16 (%)	0.908
**Age**	**18–30 years**	104 (23)	47 (20)	-	
	**31–40 years**	193 (43)	99 (43)	7 (44)	
	**41–50 years**	123 (27)	65 (29)	8 (50)	
	**51–60 years**	26 (6)	15 (7)	1 (6)	
	**≥61 years**	4 (0.9)	2 (0.9)	-	

**Sex**	**Male**	232 (52)	107 (47)	9 (56)	0.983
	**Female**	216 (48)	121 (53)	7 (44)	
	**undisclosed**	2 (0.4)	-	-	

**Race distance**	**10km**	43 (10)	19 (8)	-	0.932
	**20km**	131 (29)	66 (29)	-	
	**34km**	142 (32)	73 (32)	3 (19)	
	**50km**	134 (30)	70 (31)	13 (81)	

**Age and sex distribution of the DNF participants (n=16)**					

**Race distance**	**Female**	**Male**	**31**–**40**	**41**–**50**	**51**–**60**

34km	2	1	3	0	0
50km	5	8	4	8	1

n, number;

# p-value, all trail run entrants vs. all consenting trail runners that completed the post-race survey

**Table 2:** Anthropometric characteristics (height, weight, and BMI) and running experience by males and females in the DNF group of runners who entered and started the 34km or 50km races

Characteristics		Participants who completed both pre- and post-race surveys	All DNF participants	Female DNF participants	Male DNF participants	p-value#

		n=228	n=16	n=7	n=9	

**Anthropometric measurements mean**±**SD**	**Height (cm)**	173.2±9.7	174.6±12.2	163.9±4.3	183.0±9.2	0.001*
	**Weight (kg)**	71.1±14.1	75.5±14.9	65.4±9.2	83.3±14.0	0.009*
	**BMI (kg/m** **2)**	23.5±3.5	24.6±2.7	24.3±2.4	24.8±3.1	1.000

**Total running experience n (%)**	**0 to 2 yrs**	46 (20%)	2 (13%)	1 (14%)	1 (11.1%)	0.498
	**>2 to 5 yrs**	78 (34%)	6 (38%)	4 (57%)	2 (22%)	
	**>5 yrs**	104 (46%)	8 (50%)	2 (29%)	6 (67%)	

**Trail running experience n (%)**	**0 to 2 yrs**	112 (49%)	3 (19%)	2 (29%)	1 (11%)	0.377
	**>2 to 5 yrs**	66 (29%)	10 (63%)	5 (71%)	5 (56%)	
	**>5 yrs**	50 (22%)	3 (19%)	-	3 (33%)	

n, number; BMI, body mass index; DNF, did not finish; SD, standard deviation

# p-value; males vs females of the DNF group;

* statistical significance (p<0.05)

**Table 3:** Pre-race injury and illness history of runners who entered and started the 34km or 50km races, along with their race participation and completion status

Variable name		Participants who completed both pre- and post-race surveys	All DNF participants	Female DNF participants	Male DNF participants	p-value#

		n=228 (%)	n=16 (%)	n=7 (%)	n=9 (%)	

**Did you START the race you registered for?**	**No**	6 (3)	-	-	-	N/A
	**Yes**	222 (97)	16 (100)	7 (100)	9 (100)	

**Did you FINISH the race you registered for?**	**No**	16 (7)	16 (100)	7 (100)	9 (100)	N/A
	**Yes**	206 (90)	-	-	-	

**Previous running injury (in past 6 months)**	**No**	165 (72)	10 (63)	5 (71)	5 (56)	0.633
	**Yes**	63 (28)	6 (38)	2 (29)	4 (44)	

**Current running injury (at study entry)**	**No**	164 (72)	10 (63)	5 (71)	5 (56)	N/A
	**Yes**	2 (0.9)	Missing	Missing	Missing	

**Chronic disease**	**No**	194 (85)	15 (94)	6 (86)	9 (100)	0.438
	**Yes**	34 (15)	1 (6)	1 (14)	-	

**Current illness (at study entry)**	**No**	217 (95)	15 (94)	7 (100)	8 (89)	1.000
	**Yes**	7 (3)	1 (6)	0 (0)	1 (11)	

n, number;

# p-value, males vs females of the DNF group

**Table 4:** Athlete responses on the location, distance, and time of race termination, as well as the evacuation methods used for runners who entered and started the 34km or 50km races

At what distance (km) did you stop participating in the race? Distance originally entered for		All DNF participants	Female DNF participants	Male DNF participants	p-value#

		n=16 (%)	n=7 (%)	n=9 (%)	

**Distance stopped at**	**Race entered (n)**				
0km–9km	**-**	0 (0)	0 (0)	0 (0)	0.633
> 9km–20km	**-**	0 (0)	0 (0)	0 (0)	
> 20km–34km	50km (3), 34km (3)	6 (38)	2 (29)	4 (44)	
> 34km	50km (10)	10 (63)	5 (71)	5 (56)	

**How long have you been running when it happened? (e.g. 20 min or 3hrs into the race)**					1.000
Between 3–5 hours		2 (13)	1 (14)	1 (11)	
Between 5–7 hours		3 (19)	1 (14)	2 (22)	
Between 7–9 hours		3 (19)	1 (14)	2 (22)	
>9 hours		7 (44)	4 (57)	3 (33)	
Unspecified		1 (6)	0 (0)	1 (11)	

**How did you manage to get back to the finish line/evacuate from the racecourse?**					0.256
Picked up by race shuttle		3 (19)	2 (29)	1 (11)	
Ran 4km back to start/finish, after 34km		1 (6)	0 (0)	1 (11)	
Assisted by race partner		1 (6)	1 (14)	0 (0)	
I got evacuated by mountain rescue staff on foot/vehicle		1 (6)	1 (14)	0 (0)	
I hiked/ran back myself/ran to nearest aid station		10 (63)	3 (43)	7 (78)	

n, number;

# p-value; males vs females of the DNF group
